# Mood Disorders: From Psychopathogenesis to Treatment

**DOI:** 10.1155/2015/289508

**Published:** 2015-11-01

**Authors:** Ru-Band Lu

**Affiliations:** ^1^Department of Psychiatry, College of Medicine and Hospital, National Cheng Kung University, Tainan, Taiwan; ^2^Institute of Behavioral Medicine, College of Medicine, National Cheng Kung University, Tainan, Taiwan; ^3^Institute of Allied Health Sciences, College of Medicine, National Cheng Kung University, Tainan, Taiwan; ^4^Addiction Research Center, National Cheng Kung University, Tainan, Taiwan; ^5^Center for Neuropsychiatric Research, National Health Research Institutes, Miaoli, Taiwan

Mood disorder is a group of diagnoses in the Diagnostic and Statistical Manual of Mental Disorders (DSM) classification system where a disturbance in the person's mood is hypothesized to be the main underlying feature. In the most recent surveys, mood disorders have the highest lifetime prevalence rate and suicide risk of any psychiatric disorders. We invite investigators to contribute original research articles as well as review articles that will stimulate the continuing efforts to understand mood disorder. Preference is given to articles with a clear empirical component, whether including hypotheses testing, treatment model building, or a review of empirical work. Theoretical and speculative articles are welcomed as well for their contribution to the forming of empirically testable hypotheses. We are particularly interested in articles focusing on the novel concept of psychopathology and treatment in mood disorders.

This journal may include several hot topics: (1) “The State of the Art of the DSM-5 “with Mixed Features” Specifier”; (2) “Risk Factors for Depression in Children and Adolescents with High Functioning Autism Spectrum Disorders”; (3) “Multivariate Statistical Analysis as a Supplementary Tool for Interpretation of Variations in Salivary Cortisol Level in Women with Major Depressive Disorder”; (4) “The Pathogenesis and Treatment of Emotion Dysregulation in Borderline Personality Disorder”; (5) “Social Anxiety among Chinese People”; (6) “One-Year Follow-Up of the Effectiveness of Cognitive Behavioral Group Therapy for Patients' Depression: A Randomized, Single-Blinded, Controlled Study”; (7) “Evaluation of the Effectiveness of a Psychoeducational Intervention in Treatment-Naïve Patients with Antidepressant Medication in Primary Care: A Randomized Controlled Trial.”

In 1999, Arkiskal et al. discussed bipolar disorders in “Consensus of Bipolar Subtypes in Barcelona.” They suggest that psychiatrists and specialists should be highly concerned about the mixed features specific to bipolar disorders. In bipolar disorders, one can hope that future research will be conducted with these bipolar spectrum definitions, further validating or invalidating them, and provide better data for future clinicians to be able to use these concepts, if validated, for better clinical outcomes. One of the articles demonstrated that mixed features in bipolar disorder are frequently arguable for clinicians. The authors proposed that a diagnostic category should be preferred to a specifier and mixed states should be better as a concept of spectrum of states in DSM-5, based on their literature review.

Higher prevalence rate of comorbidity with depression in adolescents with autistic spectrum disorder was noted as well. Another article conducted very well literature review in the following domain: prevalence, explicative hypotheses and vulnerability, risk of suicide, and depressive symptoms in the comorbidity issue between depression and higher functioning autistic spectrum disorder. Therefore, in further clinical and research studies, researches would make more in-depth research possible on incidence and risk groups, symptomatological expression, differential diagnosis, duration, and prognosis and treatment of depression, as well as prevention of suicide, in the individuals with autism spectrum disorder.

It is quite complicated to analyze the relationship between treatment response and different diagnostic variables of laboratory. The linear and nonlinear statistical tools of multivariate statistical analysis could be used. Another article suggested that hierarchical cluster analysis (HCA) and principal component analysis (PCA) are useful as complementary tools for interpretation of the results obtained by laboratory diagnostic methods, using cortisol variations in major depressive disorder as a model. However, from more than thousands of review articles, there were many factors that may influence cortisol level, such as age, gender, stress, hospitalization, life cycle, menstruation cycle, and drugs. The clinician and researchers will be well controlling those confounding factors in the clinical and research use.

The generic term “emotion dysregulation” is often used to characterize a range of behavioral phenomena that are paramount in borderline personality disorder (BPD). Following a critical review of the current theories, one of the articles tries to propose a psychodynamic theory that aspires to explain all peculiarities in the course of BPD-specific emotional dyscontrol. The authors argue that the proposed theory explains the symptoms of BPD more thoroughly and inspires a parsimonious interpretation of brain imaging findings. The author draws clinical implications of the proposed theory and, accordingly, cites an efficacy study for treatment of emotion dysregulation. A very interesting item, in DSM-5 disruptive mood dysregulation disorder, belongs to major depressive disorder and episodes duration in children and adolescents. But few studies know what is going on when they become adults. Is it BPD, emotion dysregulation phenomena as a symptom of BPD, major depression, or bipolar disorders?

Social anxiety could be influenced by education and culture. It has been well studied in Western culture but unclear in others. Another article studied the relative factors in developing social anxiety, such as attachment, parenting behavioral activation, and cultural factors in Han Chinese people. The containment of social anxiety shows difference between Han Chinese and Western populations, especially in attachment, parenting, behavioral inhibition/activation, and the collectivist cultural factor-attitude toward group. The authors suggest more culturally sensitive assessment tool of social anxiety among the Han Chinese.

Cognitive behavior group therapy (CBGT) might be effective in depressive patients. Another article demonstrated a 12-session CBGT on those patients. In a single-blind randomized controlled study with a 2-arm parallel group design, eighty-one subjects were randomly assigned to intervention group (CBGT) or control group and 62 completed the study. The primary outcome was Beck Depression Inventory and Hamilton Rating Scale for Depression. The secondary outcome was automatic thoughts measured by automatic thoughts questionnaire. Both groups were evaluated at the pretest (2 weeks before), posttest (after 12 therapy sessions), and short-term (3 months), medium-term (6 months), and long-term (12 months) follow-up. All the assessments were maintained for 1 year. The results showed that CBT is effective in depression symptoms, alternative thinking, reconstructive thoughts, automatic negative thoughts, and cognitive errors.

Antidepressant (AD) with psychoeducation (PE) had been the approved treatment of choice for patients with major depressive disorder. Although psychoeducation was suggested to be effective in treating depressed patients using a randomized controlled trial design in primary care, researchers and clinicians should be more cautious about using psychoeducation as an effective treatment in depression, such as the background of leading therapist and the dynamics between therapists and members. Consequently, researchers and clinicians are suggested to renew information about mood disorders from various aspects.

